# Effectiveness and cost-effectiveness of a telemedicine programme for preventing unplanned hospitalisations of older adults living in nursing homes: the GERONTACCESS cluster randomized clinical trial

**DOI:** 10.1186/s12877-022-03575-6

**Published:** 2022-12-22

**Authors:** Caroline Gayot, Cécile Laubarie-Mouret, Kevin Zarca, Maroua Mimouni, Noelle Cardinaud, Sandrine Luce, Isabelle Tovena, Isabelle Durand-Zaleski, Marie-Laure Laroche, Pierre-Marie Preux, Achille Tchalla

**Affiliations:** 1 Laboratoire VieSanté - UR 24134 (Vieillissement, Fragilité, Prévention, E-Santé), Institut OMEGA HEALTH, Université de Limoges, Limoges, France; 2 CHU de Limoges, Pôle HU Gérontologie Clinique, 2 Avenue Martin-Luther King, Limoges, F-87042 France; 3 Unité de Recherche Clinique Et d’Innovation (URCI) en Gérontologie, CHU de Limoges, Pôle HU Gérontologie Clinique, Limoges, France; 4 DRCI-URC Eco Ile-de-France, Assistance Publique-Hôpitaux de Paris (AP-HP), Paris, France; 5 Centre d’Épidémiologie, de Bio Statistique Et de Méthodologie de La Recherche (CEBIMER), CHU de Limoges, 2 Avenue Martin-Luther King, Limoges, F-87042 France; 6 Geriatric Medicine, University of Limoges, CHU Limoges, Laboratoire VieSanté - UR 24134, Limoges, France

**Keywords:** Nursing home, Multimorbidity, Telemedicine, Telehealth, Hospitalisation, Hospital readmission, Prevention

## Abstract

**Objective:**

The GERONTACCESS trial evaluated the utility and cost-effectiveness of a gerontological telemedicine (TLM) programme for preventing unplanned hospitalisation of residents living in nursing homes (NHs) in regions lacking medical facilities and/or qualified medical providers (“medical deserts”).

**Design:**

GERONTACCESS was a 12-month, multicentre, prospective cluster-randomised trial conducted in NHs. The intervention group underwent TLM assessments every 3 months. The control group received the usual care. In both groups, comprehensive on-site assessments were conducted at baseline and the final visit. Care requirements were documented throughout the study.

**Setting and participants:**

NH residents aged ≥ 60 years with multiple chronic diseases.

**Methods:**

The study outcomes were the proportion of patients who experienced avoidable and unplanned hospitalisation, and the incremental cost savings per quality-adjusted life years from baseline to the 12-month follow-up.

**Results:**

Of the 426 randomised participants (mean ± standard deviation age, 87.2 ± 7.6 years; 311 [73.0%] women), 23.4% in the intervention group and 32.5% in the control group experienced unplanned hospitalisation (odds ratio [OR] = 0.73, 95% confidence interval [CI] 0.43 to 0.97; *p* = 0.034). Each avoided hospitalisation in the intervention group saved $US 3,846.

**Conclusions and implications:**

The results of GERONTACCESS revealed that our gerontological, preventative TLM program significantly reduced unplanned hospitalisations. This innovative intervention limited disease progression and promoted a healthy lifestyle among NH residents.

**Trial registration:**

Clinicaltrials.gov, NCT02816177, registered June 28, 2016.

## Introduction

Populations are aging worldwide; the number of people aged over 80 years will increase threefold over the next three decades [1]. Elderly people may suffer from various combinations of geriatric syndromes, disabilities, and comorbidities. Nursing home (NH) residents are particularly likely to be transferred to an emergency department (ED), which is associated with adverse events, functional decline, and death [2–5]. Hospitalisation exposes frail residents to unnecessary health risks [6, 7]. Moreover, as many as two-thirds of nursing transfers to the hospital may be avoidable [8]. One major reason for unnecessary hospital transfers is the lack of qualified physicians and advanced practice providers available to guide medical care and advance care planning in residents living NHs [9].

One motivation for improved telehealth to NHs: is if hospitalisations were reduced, then, total system cost would be reduced through preventing the most expensive service, hospitalisations. Telemedicine (TLM) provides greater access to specialist care [10]. Many studies have demonstrated the utility of TLM for monitoring chronic conditions [11], dermatological issues [12], dental health [13], and geriatric health problems [14, 15]. The GERONTACCESS primary aim was to improve care plans and prevent development of geriatric syndromes and chronic diseases decompensation in order to reduce hospital transfers. The Comprehensive Geriatric Assessment (CGA), as a validated tool, improves the outcomes of older adults [16, 17]. Our systematic, preventative geriatric TLM assessment program (GTLM) with a follow-up component provided geriatric care expertise to NHs lacking resident geriatricians. The primary objective of the GERONTACCESS study was to evaluate the utility and cost-effectiveness of a 12-month GTLM program for reducing unplanned hospitalisation of residents of NHs with limited access to geriatric expertise.

## Methods

### Study design and population

**Fig. 1:**
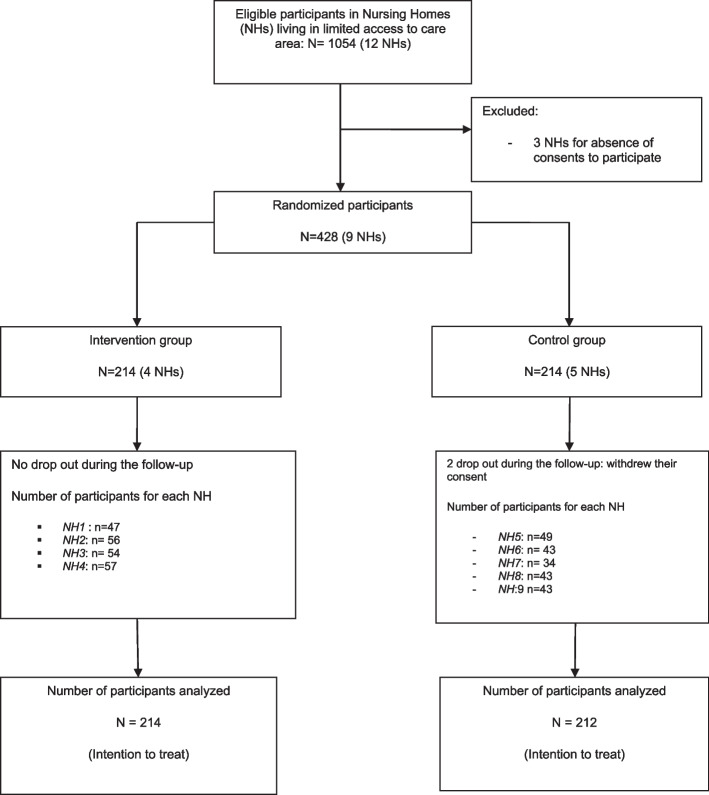
Flowchart of the GERONTACCESS study

The GERONTACCESS study, Clinicaltrials.gov, NCT02816177, registered 28/06/2016, was a prospective, multicentre, cluster-randomised, open-label trial with a control arm (usual care) and an interventional arm (GTLM program) conducted from July 2016 to January 2018 in Nouvelle Aquitaine area, France. The unit of randomisation was the NH. NHs in the intervention group implemented the GTLM program for management of multiple chronic conditions, whereas NHs in the control group managed these conditions via usual care. Nine of the twelve initially selected non-profit NHs were finally included in medical desert areas (average capacity was 77 residents (min 60, max 111)). No geriatrician was present onsite. There were four NHs in the intervention group and five in the control group. All participants have been admitted for long-term care accommodation, they were aged 60 years and over; and had at least two chronic diseases. The inclusion and follow-up procedures are shown in Fig. 1. Written informed consent was obtained from all participants or their legal representatives.

### Intervention

#### Telemedicine for the intervention group

The NHs in the intervention group received funding from the France Public Health Ministry to equip themselves with telemedicine tools as part of this experiment to optimize access to care. In accordance with French law, we used the secure TELEmedicine Aquitaine (TELEA) platform, which is specifically for the Nouvelle-Aquitaine region. TELEA ensures the security of patient and nurse data, and stores all informed consent forms and clinical files. A geriatrician can write a TLM report using the TELEA platform and send it via a secure messaging system to a physician. The equipment used during the GERONTACCESS study included a videoconferencing system, high-resolution camera (to aid wound care), mobile camera (to record residents as they walked around a room), stethoscope, electrocardiograph, and combined otoscope/dermatoscope.

#### Intervention

The intervention involved an initial teleconsultation within 10 days of inclusion. During this first teleconsultation a care plan was agreed upon by the resident, geriatrician, and NH staff and sent to attending physician. Three follow-up preventative teleconsultations were performed at 3, 6, and 9 months later with a mini-CGA to screen the geriatric syndromes and readjust the care plan as necessary. Teleconsultation were mostly conducted in early afternoon and they lasted 15 to 30 min. If necessary, the following connected devices were used. The stethoscope for a cardiac auscultation, a camera for the oral examination and sometimes the 'EKG' for an electrocardiogram for the follow-up of coronary disease or cardiac rhythm or conduction disorders. These examinations aim to limit avoidable non-programmed hospitalisations by avoiding decompensation of comorbidities. Unplanned teleconsultations could be requested by NH staff at any time. All treating physicians were at liberty to disregard the geriatrician’s advice.

### Control group

In the control NHs, residents received the usual physician care.

### Outcome measures and data collection

The primary outcome was the proportion of residents experiencing unplanned hospitalisation (defined as hospitalisation due to degeneration of a condition identified at baseline, or an emergency department admission followed by hospitalisation) during the 12-month study period. The secondary endpoint (both arms) was the number of unplanned hospitalisations (medical or surgical) during the same period. A face-to-face evaluation using the CGA was performed by the geriatrician of the mobile team at baseline and 12 months thereafter. Medico-economic data were collected every month.

### Economic evaluation

Only direct costs were assessed (as recommended by the French National Authority for Health [HAS]) [18]. The calculation method, data sources, and expenses incurred by the health insurance provider and healthcare system are shown in Tables 1 and 2. Costs and programme utility were evaluated over 1 year, and an incremental cost-effectiveness ratio (ICER) was calculated. Bootstrapping was used to quantify variability among the costs and outcomes. Furthermore, 1,000 matched estimates of the average incremental costs and outcomes in each group were plotted on a cost-effectiveness plane.

**Table 1:** Baseline characteristics of the GERONTACCESS Study population

	**Intervention group**	**Control group**	**n.**	***p*** **value**
*Demographic informations*				
**Age** (yrs), mean ± SD				
_	87.1 (± 7.58)	87.5 (± 7.42)	426	0.58
**Gender**, No. (%)				
Female	157 (73)	154 (73)	311	0.87
*Social informations*				
**Academic level**, No. (%)				
No certification	87 (44)	78 (40)	165	0.08
Certification	104 (53)	117 (59)	221	
Higher studies	6 (3)	2 (1)	8	
**Family status**, No. (%)				
Single/Divorced	37 (17)	44 (21)	81	0.71
Married	27 (13)	32 (15)	59	
Widower	147 (70)	135 (64)	282	
**Legal protection**, No. (%)				
Safeguard	0 (0)	1 (2)	1	0.85
Guardianship	17 (30)	17 (32)	34	
Tutorship	40 (70)	35 (66)	75	
**Monthly revenues**, No. (%)				
< $642	25 (18)	16 (10)	41	0.02
[$642; $1028]	57 (41)	56 (36)	113	
[$1028; $1542]	32 (23)	59 (38)	91	
> $1542	25 (18)	26 (16)	51	
**EQ-5D** No. (%)	144 (67)	149 (70)	293	0.51
**Utility,** mean ± SD	0.382 (± 0.370)	0.292 (± 0.366)	293	0.04
**Functional independence SMAF score,** mean ± SD	-45.2 (± 15.1)	-46.3 (± 15.0)	423	0.46
**Medical condition** No. (%)				
Heart rhythm disorder	52 (24)	55 (26)	107	0.90
HBP	98 (46)	124 (58)	222	0.01
Diabetes	36 (17)	44 (21)	80	0.30
Neuro-cognitive disorders	134 (63)	140 (66)	274	0.50
Depression	74 (35)	85 (40)	159	0.20
Obstructive pulmonary disease	20 (9)	20 (9)	40	1.00
Comorbidity mean/subject	5,53 (± 4.18)	5,63 (± 3.89)	426	0.60
Treatments mean/subject	10,43 (± 8.23)	11.17 (± 8.23)	426	0.35
**Hospitalization previous year** No. (%)	53 (25)	50 (24)	103	0.90
**ADL,** mean ± SD	3.33 (± 1.65)	3.30 (± 1.63)	425	0.85
**IADL,** mean ± SD	1.46 (± 1.42)	1.54 (± 1.42)	424	0.54
**Nutritional status,** MNA score mean ± SD	20.6 (± 4.08)	21.0 (± 3.87)	405	0.23
**NPI**, mean ± SD	11.7 (± 12.2)	11.6 (± 14.4)	92	0.99
**Cognitive deficit**, No. (%)				
MMSE score < 24	127 (80)	122 (77)	249	0.05
**Geriatric Depression Scale (Mini-GDS score)**, mean ± SD	1.47 (± 1.41)	1.37 (± 1.32)	255	0.54

**Table 2:** Clinical outcomes at 12 months

Clinical outcomes	Intervention group	Control group	n.	*p* value
**Participants with unplanned hospitalizations** No. (%)	50 (23)	69 (33)	119	0.034
**Number of unplanned hospitalizations** mean ± SD	0.29 (± 0.77)	0.44 (± 0.99)	154	0.17
**Length of stay in day** mean ± SD	9.37 (± 8.78)	8.14 (± 6.32)	147	0.392
**ED admissions without hospitalization** No. (%)	29 (14)	22 (10)	51	0.314
**Consultations by referring physician** mean ± SD	16.4 (± 6.94)	15.1 (± 5.55)	426	0.040
**Deaths** No. (%)	40 (19)	43 (20)	83	0.68
**EQ-5D**	83 (39)	77 (36)	160	0.60
**Utility**	0.309 (± 0.342)	0.329 (± 0.346)	160	0.73

### Sample size

We performed a superiority test; based on an alpha risk of 5%, beta risk of 10%, estimated annual hospital admission incidence of 30% [19], and 25% reduction in the risk of admission, a minimum of 388 subjects (194 per group) were required. We added a 10% margin to account for non-evaluable subjects; thus, 428 subjects were needed (214 per group). All calculations were performed using nQuery Advisor ver. 7.0 software.

### Statistical analysis

Data are presented as mean ± standard deviation (SD) or percentages, as appropriate. We used linear mixed regression models to compare quantitative outcome variables. Logistic models were used if the outcomes were binary, using patient as a fixed effect and NH as a random effect. Changes in utility were compared by analysis of covariance (ANCOVA), adjusting for the baseline and mean scores for each NH. The level of significance was set to 5%, and all analyses were performed on an intention-to-treat basis. R software (R Development Core Team, Vienna, Austria) was used for the data analysis.

## Results

**Fig. 2:**
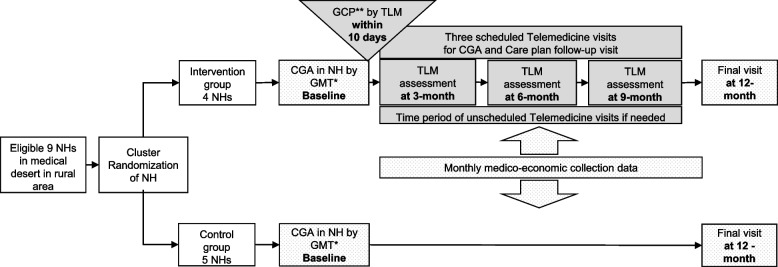
The design of the GTLM programme: GERONTACCESS study protocol. *geriatric mobile team. **gerontological care plan (formulated by the multidisciplinary geriatric mobile team staff and sent to the NH physician within 10 working days)

Of the 426 patients (Figs. 1 and 2), 214 and 212 were assigned to the intervention and control groups, respectively; 53 (25%) and 50 (24%), respectively, had been hospitalised the year before inclusion. Among the patients for whom the cost of care was evaluated, 73% were female (mean age, 87 years). Patient baseline characteristics are shown in Table 1. In terms of health insurance costs, the average total in the intervention group was $US 1,900 ± 3,040 and $US 2,250 ± 3,450 in the control group (*p* = 0.27). The total costs included consultations/teleconsultations, emergency department admissions followed by a hospitalisation and/or unplanned hospitalisations, and transportation costs. The mean number of consultations by a referring physician was 16.4 ± 6.94 in the intervention group and 15.1 ± 5.55 in the control group (*p* = 0.04). In the intervention group, 631 teleconsultations were performed during the scheduled TLM visits. Very few unscheduled teleconsultations were conducted: 2 with geriatrician and 8 with other specialists (dermalogist, psychiatrician and psychogeriatrian). The average number of TLM procedures in the intervention group was 3 ± 1.02. In terms of hospitalisation, 14% of the intervention group and 10% of the control group were admitted to emergency department without hospitalisation during the follow-up period (*p* = 0.314).

### Effectiveness analysis

The proportion of unplanned hospitalisations was 23.4% (50 residents) in the intervention group and 32.5% (69 residents) in the control group (odds ratio = 0.73; 95% confidence interval [CI] 0.43 to 0.97; *p* = 0.034). During the 12-month follow-up, 61 unplanned hospitalisations occurred in the intervention group versus 93 in the control group. The mean number of theses unplanned hospitalisations was 0.285 ± 0.563 in the intervention group and 0.443 ± 0.78 in the control group; the difference of 0.158 was not significant (*p* = 0.17). The mean number of consultations/patients during the study was 16.4 ± 6.94 in the intervention group and 15.1 ± 5.55 in the control group. Forty (19%) deaths occurred in the intervention group, compared to forty-three (20%) in the control group (*p* = 0.68).

### Cost-effectiveness

**Fig. 3:**
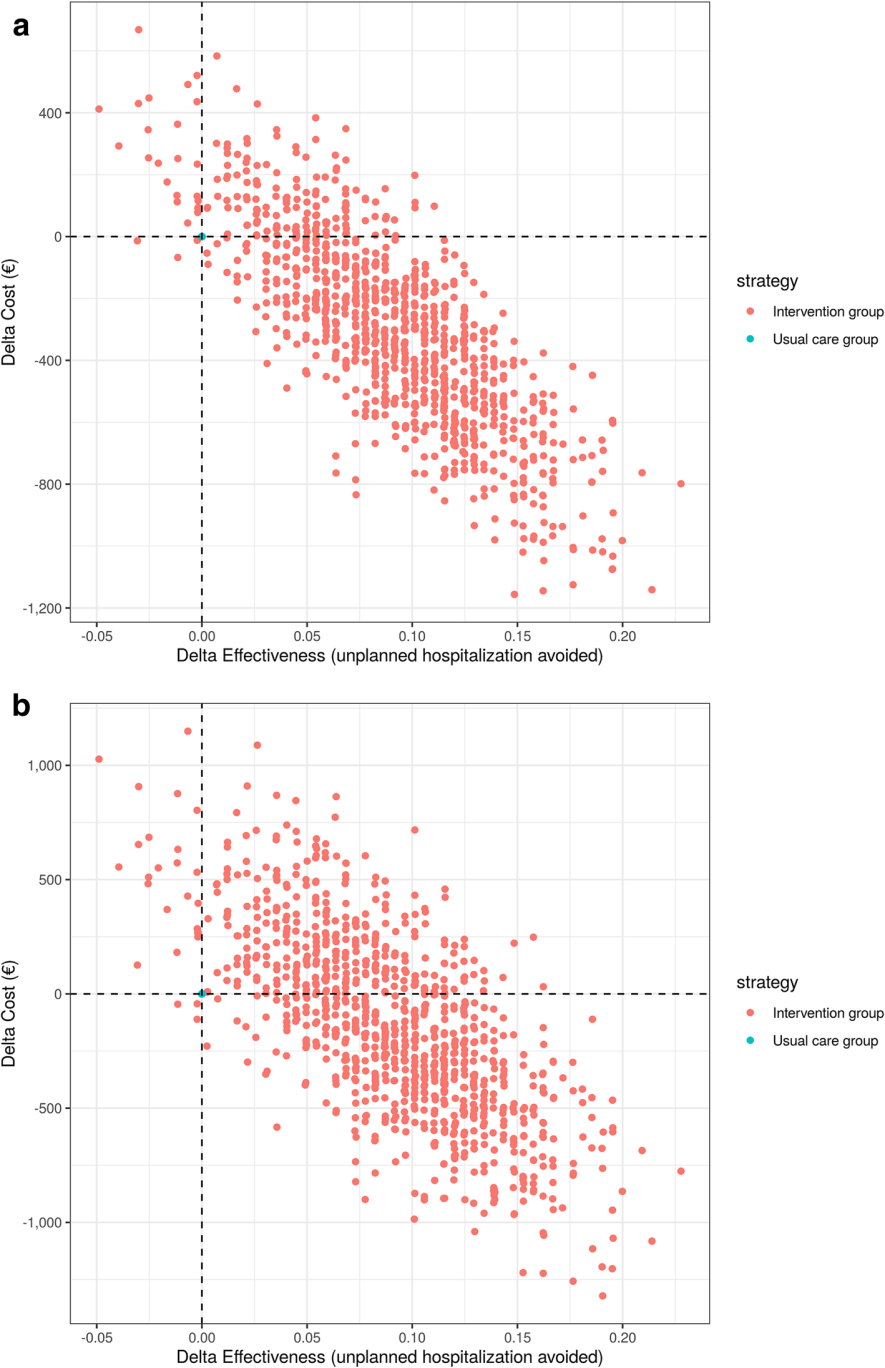
Bootstrap distribution of 1,000 ICERs ($US/unplanned hospitalisation avoided)

The incremental cost saving was $3,846 for each avoided hospitalisation in the intervention group (Tables 3 and 4). The scatterplot of the 1,000 ICERs calculated during the bootstrap analysis, indicate that 86% of the points felt within the southwestern quadrant of the cost-effectiveness plane, i.e. the intervention was dominant (less costly and less unplanned hospitalization) (Fig. 3).

**Table 3:** Detailed costs in dollars ($) in each group

	**Intervention group** ***N* = 214**	**Control group** ***N* = 212**	***p*** **value**
**Costs from the Point of view of the health insurance ($)**			
Consultations	428,01 (± 181,87)	395,33 (± 144,84)	0.04
Teleconsultations	71,88 (± 25,05)	_	_
Unplanned hospitalizations	1,235 (± 2,840)	1,610 (± 3,080)	0.19
ER admissions	65,34 (± 321,27)	105,64 (± 754,71)	0.46
Transports	101,28 (± 174,25)	137,22 (± 247,21)	0.08
**Total cost per patient**	**1,900 (± 3,040)**	**2,250 (± 3,450)**	**0.27**
**Costs from the Point of view of the care provider ($)**			
Consultations	445,42 (± 189,49)	412,75 (± 151,38)	0.04
Teleconsultations	75,14 (± 26,14)	_	_
Unplanned hospitalizations	1,570 (± 4,390)	1,780 (± 3,740)	0.59
ER admissions	80,59 (± 347,41)	131,78 (± 754,71)	0.36
Transports	116,53 (± 233,06)	144.97 (± 258,11)	0.24
**Total cost per patient**	**2,290 (± 4,600)**	**2,470 (± 4,120)**	**0.66**

**Table 4:** Total cost and effectiveness in each group; from the point of view of the health insurance and the care producer

	**Intervention group** *N* = 214	**Control group** *N* = 212	**Difference** **(Δ)**	***p *** **value**
**COST (**$)				
From the perspective of health insurance				
** Total cost 1** mean ± (SD)	1,900 (± 3,040)	2,250 (± 3,450)	- 350	0.274
From the perspective of the care producer				
** Total cost 2** mean ± (SD)	2,290 (± 4,600)	2,470 (± 4,120)	- 180	0.662
**EFFECTIVENESS**				
** The proportion of patients with unplanned hospitalization**	0.234	0.325	0.091	0.034
**ICER 1 (ΔC1/ ΔE) = **	**-3,846**			
**ICER 2 (ΔC2/ ΔE) = **	**-1,978**

## Discussion

We performed a trial to evaluate the utility and cost-effectiveness of a GTLM programme. The programme reduced the proportion of NH residents admitted to the hospital, but did not reduce the number of hospitalisations. Three important points for NH residents and policymakers emerged. First, the GTLM programme provides remote geriatric expertise. Although the CGA has been validated for use in routine geriatric care [20–22], we found that on-site administration of the CGA by a geriatrician was valuable. In contrast to assessments made prior to an emergency department transfer [23], the CGA was performed in the resident’s normal environment under stress-free conditions in this study. A holistic, personalized, and adaptable care plan was then initiated, in consultation with the NH staff tasked with implementing it. The subsequent teleconsultations evaluated geriatric syndromes every 3 months, thereby enhancing anticipatory care to help avoid unplanned hospitalisations caused by complications of chronic multimorbidities or the worsening of a condition. The proportion of residents who avoided unplanned hospitalisation was significantly greater in the intervention group even if some of them experienced multiple hospitalisations in intervention group. In this study, the rate of mortality was not significant between the two groups. Those results are similar to those observed in literature [24]. However, the GTLM programme increased general practitioner consultations and seemed to increase emergency department visits without hospitalisation probably linked to the excessive medicalization of residents. Then it was not designed to manage emergencies, and there was no significant group difference in emergency department admissions.

Due to the robustness of the study design, the evidence regarding the utility of GTLM can be considered strong. If TLM includes a primary care consultation, the likelihood of hospital transfer is reduced [25]. We found that each hospitalisation avoided in the intervention group saved Medicare costs in the amount of $US 3,846. This does not include investment in technology, because it is part of a systematic allocation that is now basically free to all NHs. The telemedicine has not generated any new costs and is included in the current care covered by the health insurance. Then, costs for technology solution for TLM acquisition has been depreciate since 2016: technology is three-fold cheaper today.

The GERONTACCESS study improved the healthcare management of NH residents with limited access to care, even though the programme included primary care visits. Geriatric prevention via TLM is less costly than degeneration of a chronic condition. By detecting early geriatric syndrome or decompensation of chronical diseases, the GTLM programme may be limits disease progression, reveals early signs of deterioration. Therefore, it should be favoured by policymakers.

### Limitations

The cost-utility of the GERONTACCESS study was not significant at 12 months, unlike many other studies [26]; this could be explained by missing data on more than 20% of the EuroQol- 5 Dimension (EQ5D) questionnaires (in turn explained by 20% of the residents being cognitively impaired). In this study we observed a high number of general practitioner consultation probably due to a contamination bias. Although TLM enhances cooperation among healthcare professionals [27], the NH nurses needed support throughout the study to use the TLM technology; TLM requires resident NH healthcare professionals, but French NHs are notoriously understaffed [28, 29]. Finally, a sociological analysis would have been useful to explore practice changes made within the NHs, as well as changes in the relationships between NHs and remote geriatricians, and in the perceptions of residents, NH’staff, geriatricians, and residents’ families. Given the novelty of this sociotechnical approach, such changes are inevitable [30, 31]. Nevertheless, we have taken the first steps towards implementation of TLM, which is critical given that populations with poor access to geriatric services are projected to grow.

## Data Availability

The datasets analysed during the current study and the study protocol are available from the corresponding author on reasonable request.
